# Age-Related Disease Burden in China, 1997–2017: Findings From the Global Burden of Disease Study

**DOI:** 10.3389/fpubh.2021.638704

**Published:** 2021-02-25

**Authors:** Dan Hu, Wu Yan, Jing Zhu, Ying Zhu, Jiaying Chen

**Affiliations:** ^1^School of Health Policy & Management, Nanjing Medical University, Nanjing, China; ^2^Institute of Healthy Jiangsu Development, Nanjing Medical University, Nanjing, China; ^3^Creative Health Policy Research Group, Nanjing Medical University, Nanjing, China; ^4^School of Public Health, Nanjing Medical University, Nanjing, China; ^5^Division of Medical Affairs, the Affiliated Jiangning Hospital of Nanjing Medical University, Nanjing, China; ^6^Respiratory and Critical Care Medicine, The Affiliated Jiangning Hospital of Nanjing Medical University, Nanjing, China

**Keywords:** age-related disease, disease burden, Chinese population, non-communicable diseases, healthy aging

## Abstract

**Background:** The population is aging much faster in China than other low- and middle-income countries. With the accelerated aging of the population, incidence and disease burden of age-related diseases have also continued to increase. Exploring the burden of age-related diseases is crucial for early disease prevention, assessing the extent of population aging, and achieving the goal of healthy aging.

**Methods:** We used the dataset from the Global Burden of Diseases, Injuries, and Risk Factors Study (GBD), and selected data on incidence, prevalence, and disease burden in China, in 1997, 2007, and 2017. We classified age-related diseases, which were defined as diseases in which the incidence rate increased quadratically with age in the adult population. Additionally, we described the changes in age-related diseases during the study period by different GBD categories. It also measured changes in the age-related disease burden in our study period, including disability-adjusted life years (DALY), years of life lost (YLL), and years lived with disability (YLD). Finally, we compared the differences in the age-related disease burdens for men and women.

**Results:** Among the 293 diseases listed in the GBD study, 69 in 2017, 78 in 1997 and 72 in 2007 were identified as age-related diseases. More than half of the age-related diseases belonged to non-communicable diseases (NCDs) in our study period. The rate of age-standardized age-related disease burden decreased between 1997 and 2017. DALYs decreased by 24.89% for non-age-related diseases and by 50.15% in age-related diseases from 1997 to 2017. The age-related disease burden of men was higher than that of women; we found a decreasing trend, with −46.23% in men and −54.90% in women.

**Conclusions:** Comparing characteristics of the aging population in China and the world, we found that China does not have the typical disease characteristics of aging society. Currently, China faces the dual threat of NCDs and communicable diseases, and NCDs account for the vast majority of the age-related disease burden. Our health systems should focus on disease prevention and early detection among the entire population, instead of treatment. Further studies should focus on reducing the duration and severity of morbidity in later life.

## Introduction

According to the report of World Health Organization (WHO), the global elderly population is expected to increase from 12 to 22% by 2050 (1). In China, the aging population is expected to grow faster than it has previously. China's elderly population (over 65 years old) is likely to rise to 330 million, or approximately one-quarter of its total population by 2050 (2, 3). It is highly probable that, as the expected human lifespan increases, these additional years are likely to be accompanied by decline in physical and mental abilities (4). With these age-related declines, the related burden will likely increase over the foreseeable future in China (5). However, not all people over 60 years of age experience such changes. Early prevention of age-related diseases can decrease the negative impact of aging and the burden of disease in the elderly population (6).

WHO has developed a new concept of healthy aging as being much more than the absence of disease (7). Based on this conceptualization, the Global Strategy and Action Plan on Aging and Health provides a political mandate for the actions required to ensure that everyone has the opportunity to enjoy a long, healthy life (8). The 2030 Agenda for Sustainable Development also makes it clear that neither a healthy life nor the right to health starts or ends at a specific age. Increasing recognition that many of the determinants of healthy aging are found in earlier life has prompted focus on how life course approaches might be used to identify critical periods during which action can be taken (9). Therefore, we should address what can be done both at younger ages and in the second half of life to improve the chances of healthy aging.

Health problems in older age go beyond what can be caused by disease alone, such as impairments in cognition, mood, and physical performance (10). Thus, many researchers (11–13) currently believe that we have reached “the end of the disease era”; however, it is possible to reduce the incidence of age-related diseases by taking early action. To prevent age-related diseases and extend the healthy and maximal lifespan, it is necessary to decrease the negative effects of aging over time (14). Many countries have begun to strengthen national capacities to formulate evidence-based policies for healthy aging. Age-related disease has recently gained increasing attention, given the growth in the older adult population (4, 14–16). More people have died in recent years from age-related diseases than ever before (17), such as cancer, hypertension, and stroke; however, these diseases are difficult to eliminate. Many studies (18–20) explored the burden of disease based on one or more age-related diseases, but there is no clear definition as to what extent the burden impacts one's overall well-being. Researchers (18, 20) have shown that glaucoma, atherosclerosis, obesity, diabetic complications, tumors, prostatic hyperplasia, Alzheimer's disease, Parkinson's disease, age-related macular degeneration, and osteoarthritis are closely related to aging, suggesting a common underlying process. Furthermore, several studies have focused on the burden of age-related disease. For example, Bani (4) analyzed the age-related disease burden in India, and found that the incidence of cardiac abnormalities was the most common cause in all age groups. Chang et al. (16) defined 92 age-related diseases, which accounted for 51.3% of the total disease burden globally based on the data from the 2017 GBD. It is crucial to identify what diseases are age-related and from there determine their disease burden. Unfortunately, there is little evidence to clarify which diseases are most associated with aging in China.

In our study, we attempted to screen out age-related diseases in the Chinese population by using GBD data, and compared the changes in the types of age-related diseases in 1997, 2007, and 2017 and analyzed the burden of age-related and non-age-related diseases during those 3 years. In this paper, to improve current understanding of the health problems faced by older adults, we analyzed the characteristics of age-related diseases in China. Furthermore, we analyzed the types and changing trends of age-related diseases to provide a basis for targeted interventions, such as early disease prevention and health management among older populations. Our findings could help clarify the sequencing and effectiveness of interventions, as well as develop design policies to support healthy aging in different contexts.

## Materials and Methods

### Data Sources

In this study, data were extracted from the GBD, which collected data from 195 countries from 1990–2017. We used the GBD interactive data visualization tool “GBD Compare” to retrieve estimates for levels and trends of disease incidence and prevalence cases, disability-adjusted life years (DALYs) and their components, years of life lost (YLLs), and years lived with disability (YLDs) for 293 diseases found in adults in China (GBD 2017 Results. Seattle, United States: Institute for Health Metrics and Evaluation, 2017; http://vizhub.healthdata.org/gbd-compare/). To compare the trends over two decades, we analyzed the related data in 1997, 2007, and 2017. The GBD study design and methods have been described in previous literature (21–23).

### GBD Causes List

In the GBD study, 293 causes of disease were organized into hierarchical levels. Level 1 contains three broad cause groups: communicable, maternal, neonatal, and nutritional diseases (CMNN), non-communicable diseases (NCDs), and injuries. Since many previous studies (16, 24) have shown age-related diseases to be mainly associated with NCDs, we divided NCDs into 12 groups, including neoplasms, cardiovascular diseases, chronic respiratory diseases, digestive diseases, neurological disorders, mental and substance use disorders, diabetes and kidney diseases, skin and subcutaneous diseases, sense organ diseases, musculoskeletal disorders and other NCDs. Thus, we have divided the age-related diseases and their related burden in the 14 GBD disease groups.

### Selection of Age-Related Diseases

In our study, we used the same approach as the previous study performed by Chang et al., to screen for age-related diseases and their burden in the Chinese population; meanwhile, we defined age-related diseases as diseases with incidence rates that increased quadratically with age among adult population (10, 16, 25–27). Adults were defined as individuals aged 25 years and older (28). To identify age-related diseases, we applied a two-step regression framework for the incidence rates using the GBD datasets for China from 1997, 2007, and 2017. A detailed description of the regression framework can be found in previous literature on age-related disease (16). For a small subset of GBD causes without incidence estimation, we used prevalence estimation under the same framework.

**Figure 1:**
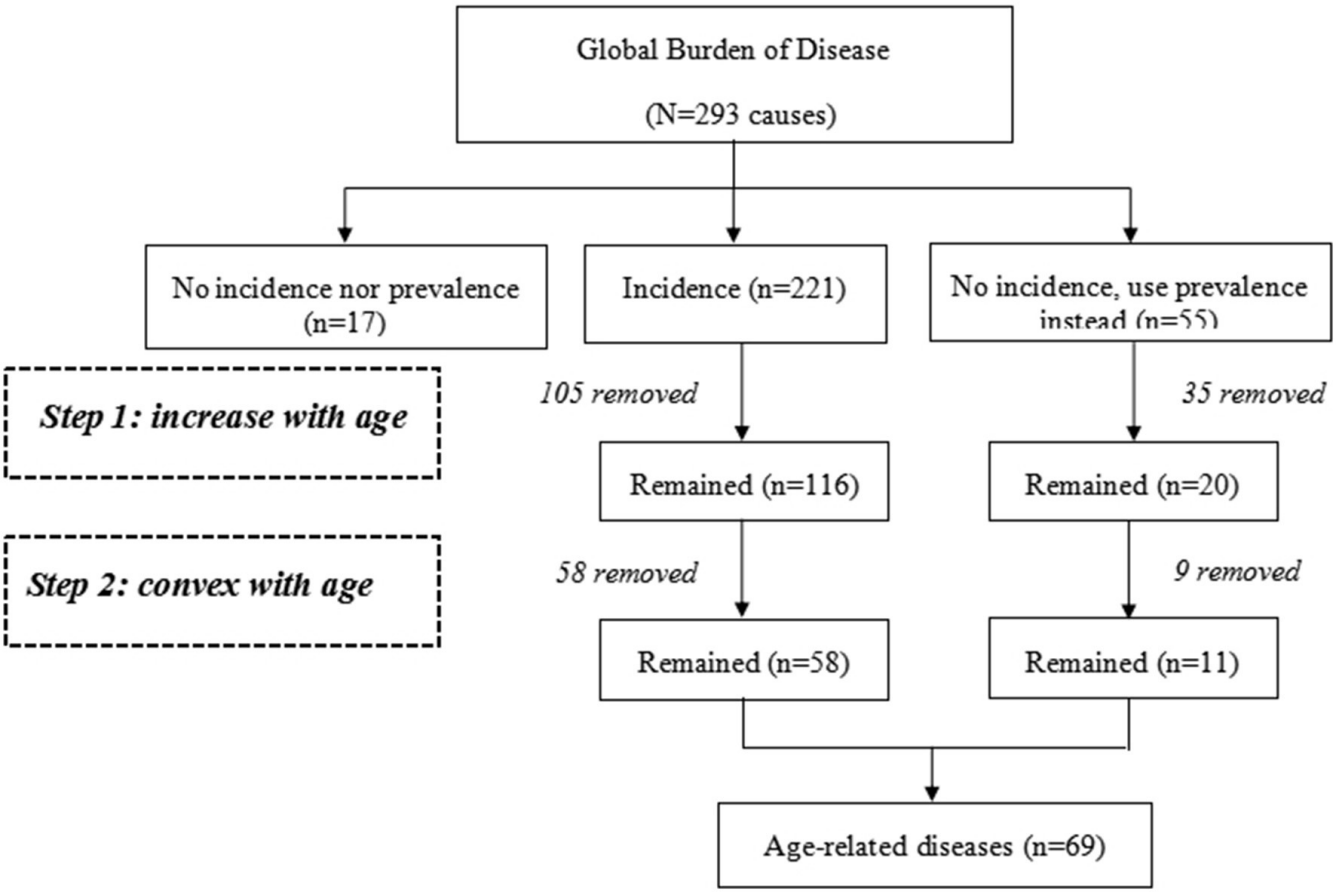
Flow chart of age-related diseases selection in 2017.

Figure 1 presents the flowchart of the selection process performed in the present study to select the data in 2017. First, we removed 17 diseases that had neither incidence nor prevalence data, and 59 diseases that did not have incidence data, from 293 GBD diseases list of China. Among the remaining 211, we excluded 106 diseases that did not show a positive relationship between incidence rates and age, and 48 that did not have a positive term for the quadratic term (i.e., the relationship between incidence rate and age was not convex). Out of the 59 causes with no incidence data, we applied the same methodology using prevalence rates, and included 11 causes that met the criteria. Overall, we identified 69 diseases associated with age.

### Defining Age-Related Disease Burden

Age-related disease burden was considered the sum of all DALYs for age-related diseases in adults. We analyzed the age-related disease burden, which is the proportion of age-related disease burden out of the total health burden. For cross-period comparisons, we also analyzed the age-standardized rates per 1,000 adults, which were adjusted for population size and age structure.

### DALYs

DALYs are a measure of overall disease burden, expressed as the cumulative number of years lost due to ill health, disability, or early death. One DALY can be thought of as one lost year of healthy life. The sum of these DALYs across the population, or the burden of disease, can be thought of as a measurement of the gap between current health status and an optimal health, in which the entire population lives to an advanced age, free of disease or disability.

DALYs for a disease or health condition are calculated by adding the YLLs due to premature mortality in the population and the YLDs for people living with the health condition or its consequences. YLLs are calculated by multiplying the number of deaths associated with the condition by the remaining life expectancy. YLDs are calculated by multiplying the number of cases with a certain health outcome by the weight of the specified disability.

### Statistical Analysis

All analyses were conducted by Stata version 12.0. To understand the relationship between age and disease prevalence at the population level, disease-specific data for both genders were summarized. Unless otherwise stated, all rates were expressed as age-standardized for the GBD reference population. This study followed the Guidelines for Accurate and Transparent Health Estimates Reporting (GATHER) 20 recommendations.

### Uncertainty Analysis

We used the same techniques found elsewhere in GBD research design to propagate uncertainty. The final estimate was calculated using an average estimate of 1,000 samples, while 95% of the uncertainty interval (UI) was determined based on the 25th and 975th bit-sorted values of all 1,000 samples.

### Sensitivity Analysis

To verify the stability of the results, we excluded incidence data for ages 80 and above for sensitivity analysis. The analysis showed that age-related disease outcomes between 25 and 80 years were consistent with those identified in the adults ages 25 and above.

## Results

### Age-Related Diseases

In 2017, 69 (23.5%) age-related diseases were identified among the 293 GBD causes; 78 (26.6%) and 72 (24.6%) were identified in 1997 and 2007, respectively. In 2017, among the 69 conditions, 14 (20.29%) were CMNNs, 7 (10.14 %) were injuries, and 48 (69.57%) were NCDs. No significant change or injury was observed in the age-related CMNNs during the study period, but the number of age-related NCDs decreased. Additionally, we also found that several emerging age-related diseases, such as other pneumoconiosis, acute glomerulonephritis, age-related macular degeneration, and near vision loss increased in 2017 (Table 1).

**Table 1:** Age-related diseases, by different GBD disease category in China, in 1997, 2007, and 2017.

**GBD category**	**1997 (China)**	**2007 (China)**	**2017 (China)**	2017 (Global)*
Communicable, maternal, neonatal, and nutritional diseases (80 diseases)	Diarrheal diseases; Lower respiratory infections; Pneumococcal meningitis; H influenzae type B meningitis; Meningococcal meningitis; Other meningitis; Encephalitis; Tetanus; Cystic echinococcosis; Trachoma; Dengue; Acute hepatitis C; Drug-susceptible tuberculosis; Multidrug-resistant tuberculosis without extensive drug resistance; Extensively drug-resistant tuberculosis (15)	Diarrheal diseases; Lower respiratory infections; Pneumococcal meningitis; H influenzae type B meningitis; Meningococcal meningitis; Other meningitis; Encephalitis; Tetanus; Trachoma; Dengue; Protein-energy malnutrition; Acute hepatitis C; Drug-susceptible tuberculosis; Multidrug-resistant tuberculosis without extensive drug resistance; Extensively drug-resistant tuberculosis (15)	Diarrheal diseases; Lower respiratory infections; Pneumococcal meningitis; H influenzae type B meningitis; Meningococcal meningitis; Other meningitis; Encephalitis; Tetanus; Trachoma; Dengue; Acute hepatitis C; Drug-susceptible tuberculosis; Multidrug-resistant tuberculosis without extensive drug resistance; Extensively drug-resistant tuberculosis (14)	Diarrhoeal diseases; encephalitis; lower respiratory infections; pneumococcal meningitis;trachoma (5)
Neoplasms (42 diseases)	Liver cancer due to hepatitis C; Prostate cancer; Colon and rectum cancer; Lip and oral cavity cancer; Gallbladder and biliary tract cancer; Pancreatic cancer; Malignant skin melanoma; Bladder cancer; Thyroid cancer; Mesothelioma; Non-Hodgkin lymphoma; Other malignant neoplasms; Non-melanoma skin cancer (squamous-cell carcinoma); Non-melanoma skin cancer (basal-cell carcinoma); Other leukemia; Myelodysplastic, myeloproliferative, and other hematopoietic neoplasms; Liver cancer due to NASH (17)	Liver cancer due to hepatitis C; Prostate cancer; Colon and rectum cancer; Lip and oral cavity cancer; Gallbladder and biliary tract cancer; Malignant skin melanoma; Testicular cancer; Bladder cancer; Mesothelioma; Other malignant neoplasms; Non-melanoma skin cancer (squamous-cell carcinoma); Non-melanoma skin cancer (basal-cell carcinoma); Myelodysplastic, myeloproliferative, and other hematopoietic neoplasms; Liver cancer due to NASH (14)	Liver cancer due to hepatitis C; Prostate cancer; Colon and rectum cancer; Gallbladder and biliary tract cancer; Malignant skin melanoma; Testicular cancer; Bladder cancer; Mesothelioma; Other malignant neoplasms; Non-melanoma skin cancer (squamous-cell carcinoma); Non-melanoma skin cancer (basal-cell carcinoma); Myelodysplastic, myeloproliferative, and other hematopoietic neoplasms; Liver cancer due to NASH (13)	Acute lymphoid leukemia; acute myeloid leukemia; benign and *in-situ* intestinal neoplasms; bladder cancer; brain and nervous system cancer; breast cancer; chronic lymphoid leukemia; chronic myeloid leukemia; colon and rectum cancer; gallbladder and biliary tract cancer; Hodgkin lymphoma; kidney cancer; larynx cancer; lip and oral cavity cancer; liver cancer due to NASH; liver cancer due to alcohol use; liver cancer due to hepatitis C; malignant skin melanoma; mesothelioma; multiple myeloma; myelodysplastic, myeloproliferative, and other hematopoietic neoplasms; non-Hodgkin lymphoma; non-melanoma skin cancer (basal-cell carcinoma); non-melanoma skin cancer (squamous-cell carcinoma); esophageal cancer; other benign and *in-situ* neoplasms; other leukemia; other malignant neoplasms; ovarian cancer; pancreatic cancer; prostate cancer; stomach cancer; thyroid cancer; tracheal, bronchus, and lung cancer; uterine cancer (29)
Cardiovascular diseases (17 diseases)	Rheumatic heart disease; Ischemic heart disease; Ischemic stroke; Intracerebral hemorrhage; Subarachnoid hemorrhage; Hypertensive heart disease; Endocarditis; Myocarditis; Other cardiomyopathy; Non-rheumatic calcific aortic valve disease; Non-rheumatic degenerative mitral valve disease; Other non-rheumatic valve diseases (12)	Ischemic heart disease; Ischemic stroke; Intracerebral hemorrhage; Hypertensive heart disease; Endocarditis; Myocarditis; Other cardiomyopathy; Non-rheumatic calcific aortic valve disease; Non-rheumatic degenerative mitral valve disease; Other non-rheumatic valve diseases (10)	Ischemic heart disease; Ischemic stroke; Intracerebral hemorrhage; Hypertensive heart disease; Endocarditis; Myocarditis; Other cardiomyopathy; Non-rheumatic calcific aortic valve disease; Non-rheumatic degenerative mitral valve disease; Other non-rheumatic valve diseases (10)	Atrial fibrillation and flutter; endocarditis; hypertensive heart disease; intracerebral hemorrhage; ischaemic heart disease; ischaemic stroke; myocarditis; non-rheumatic calcific aortic valve disease; non-rheumatic degenerative mitral valve disease; other cardiomyopathy; other cardiovascular and circulatory diseases; other non-rheumatic valve diseases; peripheral artery disease (13)
Chronic respiratory diseases (8 diseases)	Chronic obstructive pulmonary disease; Asbestosis; Interstitial lung disease and pulmonary sarcoidosis (3)	Chronic obstructive pulmonary disease; Asbestosis (2)	Chronic obstructive pulmonary disease; Other pneumoconiosis (2)	Asbestosis; chronic obstructive pulmonary disease; coal worker pneumoconiosis; interstitial lung disease and pulmonary sarcoidosis; other pneumoconiosis; silicosis (6)
Digestive diseases (16 diseases)	Cirrhosis and other chronic liver diseases due to other causes; Peptic ulcer disease; Appendicitis; Paralytic ileus and intestinal obstruction; Vascular intestinal disorders; Gallbladder and biliary diseases; Pancreatitis; Cirrhosis due to NASH (8)	Cirrhosis and other chronic liver diseases due to other cause; Peptic ulcer disease; Appendicitis; Paralytic ileus and intestinal obstruction; Vascular intestinal disorders; Gallbladder and biliary diseases; Pancreatitis; Cirrhosis due to (8)	Peptic ulcer disease; Appendicitis; Paralytic ileus and intestinal obstruction; Vascular intestinal disorders; Gallbladder and biliary diseases; Pancreatitis (6)	Cirrhosis due to NASH; pancreatitis; paralytic ileus and intestinal obstruction; peptic ulcer disease; vascular intestinal disorderss (5)
Neurological disorders (8 diseases)	Alzheimer's disease and other dementias; Multiple sclerosis; Other neurological disorders (3)	Alzheimer's disease and other dementias; Multiple sclerosis; Other neurological disorders (3)	Alzheimer's disease and other dementias; Multiple sclerosis;Other neurological disorders (3)	Alzheimer's disease and other dementias; motor neuron disease; Parkinson's disease (3)
Mental and substance use disorders (18)	N/A	N/A	N/A	N/A
Diabetes and kidney diseases (8 diseases)	Chronic kidney disease due to glomerulonephritis; Chronic kidney disease due to other and unspecified causes (2)	Chronic kidney disease due to glomerulonephritis; Chronic kidney disease due to other and unspecified causes (2)	Acute glomerulonephritis; Chronic kidney disease due to glomerulonephritis; Chronic kidney disease due to other and unspecified causes (3)	Chronic kidney disease due to glomerulonephritis; Chronic kidney disease due to other and unspecified causes (2)
Skin and subcutaneous diseases (15 diseases)	Cellulitis; Pyoderma; Fungal skin diseases; Decubitus ulcer (4)	Cellulitis; Pyoderma; Fungal skin diseases; Decubitus ulcer (4)	Cellulitis; Pyoderma; Fungal skin diseases; Decubitus ulcer (4)	Cellulitis; decubitus ulcer; fungal skin diseases; other skin and subcutaneous diseases; pyoderma (5)
Sense organ diseases (8 diseases)	Glaucoma; Cataract; Age-related macular degeneration; Other vision loss (4)	Glaucoma; Cataract; Other vision loss (3)	Glaucoma; Cataract; Age-related macular degeneration; Other vision loss; Near vision loss (5)	Age-related and other hearing loss; age-related macular degeneration; cataract; glaucoma; other sense organ diseases; other vision loss; refraction disorders (7)
Musculoskeletal disorders (6 diseases)	N/A	N/A	N/A	N/A
Other NCD (37 diseases)	Urinary tract infections; Neural tube defects;G6PD trait; Endocrine, metabolic, blood, and immune disorders (4)	Urinary tract infections; Endocrine, metabolic, blood, and immune disorders; G6PD trait (3)	Urinary tract infections; G6PD trait (2)	Congenital musculoskeletal and limb anomalies; digestive congenital anomalies;endocrine, metabolic, blood, and immune disorders; other haemoglobinopathies and haemolytic anaemias (4)
Injuries(29 diseases)	Other transport injuries; Falls; Drowning; Self-harm by firearm; Self-harm by other specified means; Environmental heat and cold exposure (6)	Other transport injuries; Falls; Drowning; Non-venomous animal contact; Other unintentional injuries; Self-harm by firearm; Self-harm by other specified means; Environmental heat and cold exposure (8)	Other transport injuries; Drowning; Non-venomous animal contact; Other unintentional injuries; Self-harm by firearm; Self-harm by other specified means; Environmental heat and cold exposure (7)	Drowning; environmental heat and cold exposure; falls; foreign body in other body part; other transport injuries; other unintentional injuries (6)
Total age-related diseases	78 diseases	72 diseases	69 diseases	92 diseases

* *The resource come from the study performed by Chang et al. (16)*.

### Age-Related Disease Burden

A total of 248.34 DALYs were due to the age-standardized, age-related disease burden; however, 400.41 DALYs were caused by non-age-related disease. In terms of proportion, age-related disease burden accounted for 38.28% (248.34/648.75) of the total disease burden. Causes of non-age-related disease burden included neoplasms (19.91%), injuries (13.12%), neurological disorders (11.91%), CMNNs (11.59%), and musculoskeletal disorders (10.39%). For age-related diseases, the burden was primarily due to cardiovascular disease (44.16%), other NCDs (12.71%), chronic respiratory disease (12.01%), injuries (7.44%), and neoplasms (6.72%). The burden of age-related disease was greater in the cardiovascular, chronic respiratory, and other chronic disease groups than in the non-age-related group (Table 2).

**Table 2:** Distribution of non-age-related burden and age-related burden by different GBD category in China, in 2017, measured in DALYs.

**GBD category**	**Non-age-related disease**		**Age-related disease**		**All disesaes**
	**Age-standardized DALY rate (per 1,000)**	**Proportion among all (%)**	**Age-standardized DALY rate (per 1,000)**	**Proportion among all (%)**	**Age-standardized DALY rate (per 1,000)**
Communicable, maternal, neonatal, and nutritional diseases	46.41(33.74, 64.43)	11.59	18.71 (16.09,22.55)	6.59	65.12 (49.83,86.99)
Neoplasms	79.68 (71.88,86.34)	19.91	19.09 (16.86,21.56)	6.72	98.77 (88.74,107.90)
Cardiovascular diseases	11.62 (9.76,13.48)	2.90	125.45 (115.55, 133.81)	44.16	137.07 (125.30,147.29)
Chronic respiratory diseases	4.43 (3.19,6.01)	1.11	34.13 (31.93,36.97)	12.01	38.55 (35.11,42.98)
Digestive diseases	14.66(11.34,19.83)	3.66	3.98(3.49, 4.87)	1.40	18.63(14.82,24.69)
Neurological disorders	17.68(11.87,25.37)	4.42	13.74 (12.52,15.09)	4.84	31.42(24.39,40.46)
Mental and substance use disorders	47.68(32.72,65.49)	11.91	0.00(0.00,0.00)	0.00	47.68(32.72,65.49)
Diabetes and kidney diseases	20.27(15.75,25.43)	5.06	3.67 (3.16,4.20)	1.29	23.94(18.90,29.63)
Skin and subcutaneous diseases	14.59(8.67,22.92)	3.64	1.53(3.15,0.69)	0.54	16.12(9.36,26.08)
Sense organ diseases	17.49(11.78,24.59)	4.37	6.55(3.79,10.52)	2.31	24.05(15.57,35.48)
Musculoskeletal disorders	41.60(28.55,58.89)	10.39	0.00(0.00,0.00)	0.00	41.60(28.55,58.89)
Other NCD	31.66(23.32,43.47)	7.91	36.11(32.20,41.95)	12.71	67.77(55.52,85.42)
Injuries	52.53(43.50,61.43)	13.12	21.13(19.29,23.50)	7.44	73.66(62.80,84.94)
All causes	400.41(306.13,518.21)	100	248.34(223.72,276.66)	100	648.75(529.85,794.87)

In 2017, the burden of disease in China was 648.81 DALYs, of which 59.40% were caused by death and 40.60% by disability (Table 3). YLLs, YLDs, and DALYs showed a gradual decline from 1997 to 2017. The non-age-related disease DALYs decreased by 24.89% from 1997 to 2017. Furthermore, 50.15% reduction occurred in age-related diseases burden.

**Table 3:** The non-age-related burden and age-related burden by different burden indicator in China, in 1997, 2007 and 2017.

**Category**	**YLLs**			**YLDs**			**DALYs**		
	**1997**	**2007**	**2017**	**1997**	**2007**	**2017**	**1997**	**2007**	**2017**
Non-age-related group	314.17	233.70	181.76	219.03	224.03	218.72	533.2	457.73	400.48
Age-related group	448.59	261.21	203.62	49.64	43.38	44.73	498.23	304.59	248.35
Total	762.76	494.91	385.38	268.67	267.41	263.45	1,031.43	762.32	648.83
Proportion of age-related burden among all burden(%)	58.81	52.78	52.84	18.48	16.22	16.98	48.30	39.96	38.28

The data of different years were segregated separately for gender. The disease burden of men was higher than women. From 1997 to 2017, the proportion of age-related disease burden in all populations showed decreasing trend (−46.23%), which was more apparent among women (−54.90%) (Table 4).

**Table 4:** The change of non-age-related burden and age-related burden by different sex in China, in 1997–2017.

**Sex**	**Year**	**Age-related burden rate(per 1,000 adults)**	**Non-age-related burden rate(per 1,000 adults)**	**All disease burden rate(per 1,000 adults)**	**Proportion of age-related burden among all burden (%)**	**Percentage change in Age-related burden 1997–2017(%)**
Male	1997	182.92 (162.30,200.48)	190.67 (149.43,242.61)	373.60 (311.74,443.09)	48.96	−46.23
	2007	119.03(110.59,127.73)	163.94(131.04,204.87)	282.98(241.64,332.61)	42.06	
	2017	98.35(89.09,108.88)	145.11(113.17,183.91)	243.46(202.26,292.80)	40.40	
Female	1997	149.29(129.40,165.29)	164.65(124.53,218.30)	313.95(253.94,383.60)	47.55	−54.90
	2007	84.49(77.82,91.63)	141.15(107.58,184.32)	225.64(185.41,275.96)	37.44	
	2017	67.33(59.54,76.47)	121.86(89.98,162.52)	189.19(149.52,239.00)	35.59	

## Discussion

Our study showed that the number of age-related diseases decreased from 1997 to 2017, with more than half classified as NCDs, such as cardiovascular disease, chronic respiratory disease, and other NCDs. Over the past two decades, the disease burden has declined in China, especially for age-related diseases (50.15%). The burden of age-related NCDs was higher than CMNNs and injuries in 2017. Additionally, men had a higher disease burden than women.

The number of age-related diseases was less in China (69 diseases) than globally based on the results of a study performed by Angela Chang and colleagues (92 diseases) (16) in 2017, as was the proportion of age-related NCDs. This may be due to different disease conditions in China and the rest of the world. China entered became an aging society in only 18 years (1981–1999), and the aging population is growing faster than ever. From 1990 to 2020, the world's elderly population grew at an average annual rate of 2.5%, compared with 3.3% in China over the same period (30–32). The rapid aging process in China is also the main reason for the discrepancy between China and the world adult disease spectrum. Besides, China was recently faced with the double challenge of infectious and chronic diseases; current trends indicating a transition from a higher prevalence of CMNNs to higher numbers of NCDs (5, 33). The patients, especially with chronic diseases, are currently getting younger in China, which is also the main reason for the decrease in the number of age-related diseases. For example, the prevalence of hypertension and diabetes increased significantly, but the onset age was younger.

Our main finding was that age-standardized age-related disease burden decreased from 2007 to 2017. On one hand, it may be related to the decrease of age-related diseases during the study period. On the other hand, with the development of economy and society, people's health improved, the decrease of overall disease burden is not surprising. Whereas, one of the serious problems associated with population aging is the increasing burden of NCDs, and the prevalence of NCDs will increase along with the increasing disease burden. According to current projections, China's rapidly aging population is expected to increase the burden of NCDs by at least 40% by 2030 (3, 34). Thus, more resources should be devoted to primary health care in China, which would likely make for a more efficient health care system (34).

The burden of age-related disease was greater in the cardiovascular, chronic respiratory, and other chronic disease groups; those diseases need better predictors of their occurrence. It is crucial to study an intervention on how to block the causal mechanisms at an early stage. Published studies have shown that only about 25% of our lifespan is determined by our genes; the other 75% is determined by our lifestyles and the choices we make every day (2, 35, 36). We should therefore pay more attention to practicing a healthy lifestyle, including increased exercise and reduction in food intake and obesity. Furthermore, physiological aging is inevitable; however, pathological aging can be prevented or delayed. However, until now, the mechanisms underlying the causes of aging have not been thoroughly understood.

The disease burden and proportion of age-related burden among all burdens was lower for women than for men, and the disease burden decreased more for women than men during our study period. The contribution to the disease burden between men and women was unequal, due to the higher prevalence of chronic disease risk factors in men (29). In addition, the China Women's Development Foundation introduced screenings for gynecological diseases, and the screening rate for common diseases among women reached 75.5% in 2018. This can help strengthen public awareness of the prevention and treatment of common diseases and enhance women's capacity for self-efficacy in healthcare.

In the absence of population level aging metrics that inform longevity, health status and disease severity in China, based on the GBD database, this is the first study to explore the age-related disease burden among Chinese adults. Furthermore, we have found changes in the overall burden of age-related disease over 20 years in China. Our study has several limitations. First, while the GBD list of diseases is comprehensive, we may have missed including some diseases that are important to China's aging population. Second, we used the same methods as Chang and colleagues (16) to identify which diseases are associated with age. The difference between China and the rest of the world could lead to different results. However, as shown in our analysis, we characterized the aging of the Chinese population, which is meaningful for providing a public health framework for actions that can be taken to promote healthy aging. Third, we did not consider the influence of multimorbidity on disease burden. Many people have multiple chronic diseases at the same time (37); however, our study was meant to draw attention to the age-related disease burden, rather than disease interactions.

## Conclusion

Identifying age-related diseases is a prerequisite for providing promising therapeutic strategies to promote healthy aging in the future. Comparing the characteristics of the aging population in China and the world, we found that China does not have the typical disease characteristics of an aging society. Currently, China faces the dual threat of NCDS and communicable diseases, and NCDs account for the vast majority of the age-related disease burden. Our health systems should focus on disease prevention and early detection among the entire population, instead of treatment. Further studies should focus on reducing the duration and severity of morbidity in later life.

## Data Availability Statement

The datasets presented in this study can be found in online repositories. The names of the repository/repositories and accession number(s) can be found at: http://ghdx.healthdata.org/gbd-results-tool.

## Author Contributions

DH and JC contributed to the conception and design of the study. WY, JZ, and DH conducted the data reduction and analyses. YZ helped validate the disease screening. DH wrote the manuscript, guided the whole process, and reviewed the manuscript. All authors read and approved the manuscript before submission.

## Conflict of Interest

The authors declare that the research was conducted in the absence of any commercial or financial relationships that could be construed as a potential conflict of interest.
